# eHSP90α in front-line therapy in EGFR exon 19 deletion and 21 Leu858Arg mutations in advanced lung adenocarcinoma

**DOI:** 10.1186/s12885-024-12573-3

**Published:** 2024-07-12

**Authors:** Yingzhen Bian, Haizhou Liu, Jinglei Huang, Zhaorong Feng, Yanyan Lin, Jilin Li, Litu Zhang

**Affiliations:** 1 Department of Research, Guangxi Medical University Cancer Hospital, Guangxi Medical University, Guangxi Zhuang Autonomous Region, Nanning, 530021 China; 2 Guangxi Cancer Molecular Medicine Engineering Research Center, Guangxi Zhuang Autonomous Region, Nanning, 530021 China

**Keywords:** eHSP90α, Exon 19 deletion, Exon 21 Leu858Arg, Lung adenocarcinoma, Biomarker

## Abstract

**Purpose:**

Extracellular heat shock protein 90 AA1(eHSP90α) is intricately linked to tumor progression and prognosis. This study aimed to investigate the difference in the value of eHSP90α in post-treatment response assessment and prognosis prediction between exon 19 deletion(19DEL) and exon 21 Leu858Arg(L858R) mutation types in lung adenocarcinoma(LUAD).

**Methods:**

We analyzed the relationship between the expression of eHSP90α and clinicopathological features in 89 patients with L858R mutation and 196 patients with 19DEL mutation in LUAD. The Kaplan-Meier survival curve was used to determine their respective cut-off values and analyze the relationship between eHSP90α expression and the survival time of the two mutation types. The area under the curve (AUC) was used to evaluate the diagnostic performance of biomarkers. Then, the prognostic model was developed using the univariate-Cox multivariate-Cox and LASSO-multivariate logistic methods.

**Results:**

In LUAD patients, eHSP90α was positively correlated with carcinoembryonic antigen(CEA), carbohydrate antigen 125(CA125), and carbohydrate antigen 153(CA153). The truncated values of eHSP90α in L858R and 19DEL patients were 44.5 ng/mL and 40.8 ng/mL, respectively. Among L858R patients, eHSP90α had the best diagnostic performance (AUC = 0.765), and higher eHSP90α and T helper cells(Th cells) expression were significantly related to shorter overall survival(OS) and worse treatment response. Also, high eHSP90a expression and short progression-free survival(PFS) were significantly correlated. Among 19DEL patients, CEA had the best diagnostic efficacy (AUC = 0.734), and CEA and Th cells were independent prognostic factors that predicted shorter OS. Furthermore, high CA125 was significantly associated with short PFS and poor curative effect.

**Conclusions:**

eHSP90α has a better prognostic value in LUAD L858R patients than 19DEL, which provides a new idea for clinical diagnosis and treatment.

**Supplementary Information:**

The online version contains supplementary material available at 10.1186/s12885-024-12573-3.

## Introduction

Lung cancer has been shown to have the highest cancer-related mortality rate of all cancers [1]. Furthermore, the proportion of lung adenocarcinoma (LUAD) is the largest and still increasing [2]. Lung cancer treatment mainly includes surgical, chemotherapy, radiotherapy, and immunotherapy [3]. With the significant increase in lung cancer pathogenesis research and precision therapy, molecular targeted therapy based on lung cancer driver mutations has dramatically enhanced the overall survival(OS) rate of patients [4]. Epidermal growth factor receptor (EGFR) mutations occur mainly in LUAD. EGFR tyrosine kinase inhibitor (EGFR-TKI) sensitizing mutations are most commonly found in exon 19 deletion (19DEL) or exon 21 Leu858Arg (L858R) [5]. Moreover, some studies have found differences in the efficacy of targeted therapy for different EGFR mutation types, with some mutations being more responsive to treatment than others [6, 7]. This difference is crucial for LUAD patients’ precise treatment and prognosis.

The heat shock protein 90 (HSP90) family comprises a highly conserved chaperone protein with a molecular weight of approximately 90 kDa, widely distributed in mammals. HSP90 has different isoforms in various intracellular spaces, with heat shock protein 90 AA1(HSP90α) and heat shock protein 90 AB1(HSP90β) primarily distributed in the cytoplasm. HSP90α is the stress-inducible type, and HSP90β is expressed constitutively [8–10]. Besides its intracellular localization, HSP90α can also be excreted into the extracellular environment, referred to as extracellular HSP90α (eHSP90α). As a chaperone protein, HSP90α has numerous client proteins and is therefore involved in many critical activities, such as inflammation regulation, apoptosis, and immunity [11]. Previous research found that the expression of eHSP90α is higher in cancer patients than in healthy populations, making it a reliable prognostic marker for several cancers [12]. For instance, Han et al. demonstrated that eHSP90α could be an effective diagnostic biomarker for liver cancer and predict patient response to surgery [13]. Similarly, in lung cancer(LC), Shi et al. found it can be a valid diagnostic biomarker and can expect the response to chemotherapy [14]. Besides, Huang et al. found that high expression of eHSP90α is associated with the poor efficacy of chemotherapy and prognosis in Small Cell Lung Cancer(SCLC), and the area under the diagnosis curve of eHSP90α against SCLC appears to be 0.791, with outstanding sensitivity and specificity [15].

However, we have yet to find any studies exploring whether eHSP90 is different under different EGFR mutations in LUAD. This study aims to use eHSP90α to predict the prognosis of patients with 19DEL and L858R mutations in LUAD patients and explore its different prognostic values. In addition, we further combined eHSP90α with other serum markers to develop a predictive nomogram model to assess prognosis accurately.

## Methods

### Patient inclusion and exclusion criteria

**Fig. 1:**
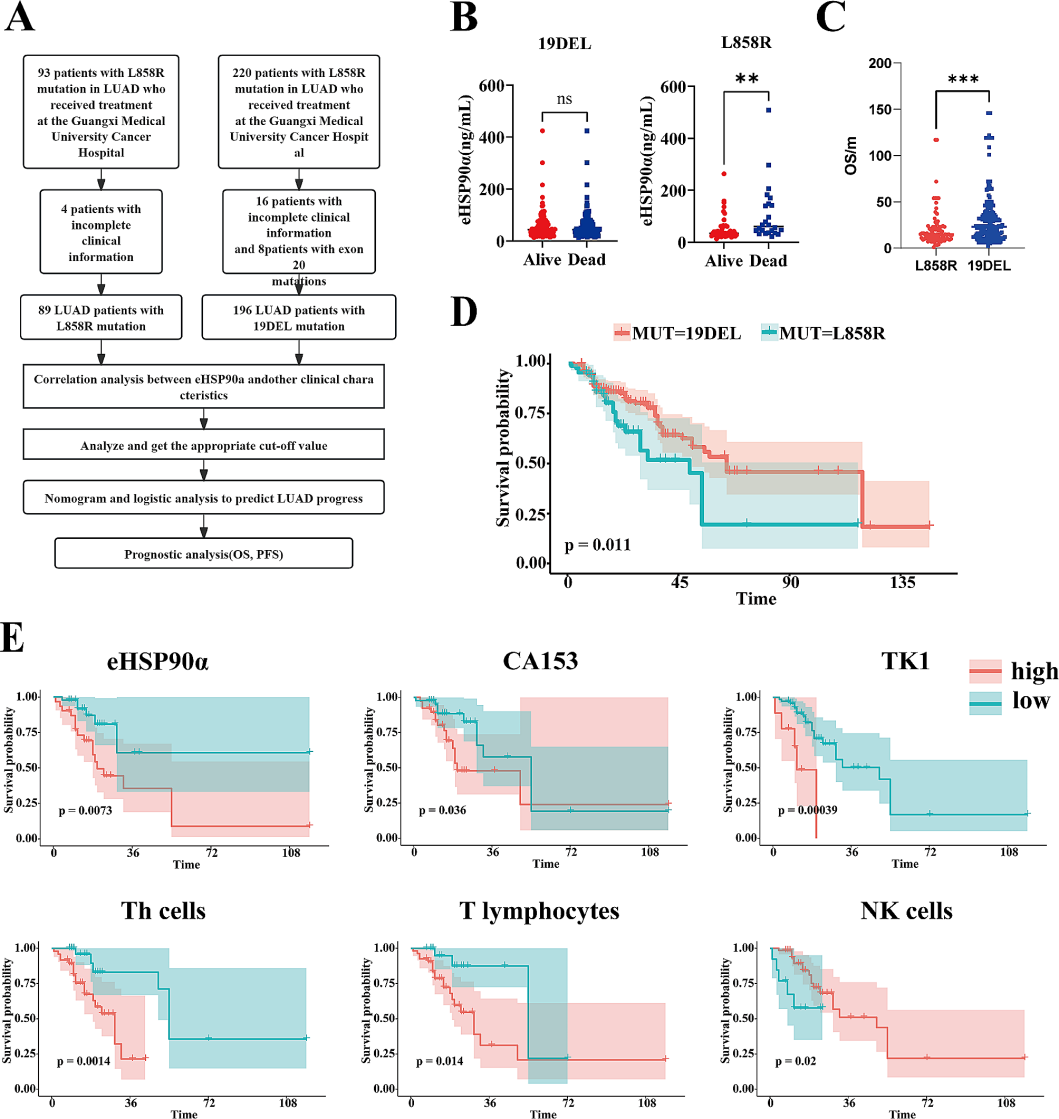
Workflow diagram and survival curves of eHSP90a expression differences, survival in lung adenocarcinoma (LUAD) EGFR exon 19 Deletion(19DEL) and 21 Leu858Arg(L858R) mutations patients, and Kaplan-Meier Survival curve of eHSP90a and overall survival(OS) in patients with LUAD L858R mutation. (**A**) Workflow. (**B**) The scatterplot shows the difference in expression of the continuous variable eHSP90a in the OS outcome state of the 19del and L858R mutations. (**C**) The scatterplot shows the OS of 19DEL and L858R mutation. (**D**) Survival of 19DEL and L858R mutation patients. (**E**) Kaplan-Meier Survival curve of eHSP90a and OS in patients with LUAD L858R mutation

This study included 93 patients with L858R in LUAD and 220 patients with 19DEL mutation admitted to the Department of Respiratory Oncology, Guangxi Medical University Cancer Hospital from July 2008 to July 2021. Four L858R patients and 16 19DEL patients were excluded due to incomplete clinical information. Eight 19DEL patients with exon 20 mutations were also excluded (Fig. 1A).

The inclusion criteria for this study were as follows: (1) Pathological diagnosis of LUAD; (2) Obtained blood samples; (3) Confirmed EGFR mutations of 19DEL or L858R only; (4) Availability of tumor markers and immune cells after admission. (5) Response assessments were conducted using chest and abdominal computed tomography scans (CT scans) every two treatment cycles, following the Response Evaluation Criteria in Solid Tumors (RECIST version 1.1). Each patient’s response was categorized into one of the following groups: responders, which encompassed cases of complete response (CR), partial response (PR), and stable disease (SD), and non-responders, which included instances of disease progression (PD). Exclusion criteria for this study were: (1) Incomplete clinical information such as tumor-node-metastasis (TNM) stage; (2) They also carried other EGFR mutations or have not been tested for EGFR gene mutations; (3) Patients who also had other oncological diseases.; (4) Incomplete follow-up information (Table 1).

**Table 1:** Inclusion and exclusion criteria

Inclusion criteria	Exclusion criteria
They have pathologically diagnosed LUAD.	Clinical information such as TNM stage was incomplete.
Blood samples could be obtained.	They also carried other EGFR mutations or has not been tested for EGFR gene mutations.
19DEL or L858R was the only EGFR mutation they carried.	Patients who also had other oncological diseases.
Tumor markers and immune cells were availabile.	Follow-up information was incomplete.
Response assessments were conducted using chest and abdominal computed tomography scans (CT scans) every two treatment cycles, following the Response Evaluation Criteria in Solid Tumors (RECIST version 1.1).	

Our outcome overall survival(OS) was based on all causes of death, while progression-free survival(PFS) was based on time for people who relapsed. Follow-up for all participants ended in July 2021. Among them, the median follow-up time of L858R mutation patients was 15 months, and for 19DEL mutation patients, it was 29 months.

### Information collection

This prospective study collected peripheral blood data of patients diagnosed with LUAD who were detected as 19DEL or L858R mutation, including age, gender, TNM staging, carbohydrate antigen 125 (CA125), carbohydrate antigen 153 (CA153), carbohydrate antigen 199 (CA199), carcinoembryonic antigen (CEA). In addition, immune cells, T lymphocytes (T cell), T helper cells (Th), suppressor T cells (Ts), T helper cells/ suppressor T cells (Th/Ts), natural killer (NK) cells and B lymphocytes (B cell) were included. All blood samples were collected from patients for the first time after admission.

### Detection of serum HSP90α

HSP90α was measured using a Yantai Protgen Biotechnology Development Co., Ltd., Shandong, China assay kit. Patient blood samples, collected in EDTA anticoagulant tubes in the early morning, were centrifuged at 3000 rpm for 15 min at 4 °C to remove particles. The assay kit was equilibrated at 37 °C for 30 min before use. Plasma samples were diluted 20 times with the diluent. Each assay plate well received 50µL of standard, plasma sample, and HSP90α marker solution. Incubating the plate at 37 °C for 60 min. After incubation, the wells were washed multiple times with the washing solution. Color developers A and B were added sequentially, and the plate was incubated at 37 °C in the dark for 20 min. The color development was then stopped by adding a stop solution. The optical density (OD) values were measured at 450 nm/620 nm within 10 min of color development termination. The HSP90α content in plasma samples was calculated based on these OD values.

### Nomogram, lasso regression, logistic regression, and cox regression

The nomogram consists of a set of parallel lines, each representing a variable and a scale along each line. Lasso Regression is a type of linear regression that uses regularization methods to prevent overfitting. In traditional linear regression, the model tries to fit the data as closely as possible, which can lead to overfitting and poor performance on new data. Logistic regression is defined as: p = \frac {1} {1 + e^{-z}}. where ‘p’ is the predicted probability, ‘z’ is the weighted sum of the input features, and ‘e’ is the mathematical constant of approximately 2.71828. The Cox regression model estimates the hazard ratio, which is the ratio of the hazard rates between two groups while adjusting for other covariates that may affect the outcome.

### Statistical analysis

Data analysis was performed using SPSS25.0 software. Clinical baseline data were presented as medians and interquartile ranges. Spearman rank correlation analysis was used to analyze the correlation between eHSP90α and other clinical biomarkers. The ROC curve was used to evaluate the diagnostic efficacy of all included clinical indicators for the prognosis of the disease. LASSO and multivariate logistic regression analyses were used to analyze the relationship between response to treatment and eHSP90α. Univariate and multivariate Cox regression analyses analyzed the relationship between eHSP90α and OS and PFS. Peripheral blood indicators less than 0.05 were included in the multivariate analysis. The best cut-off value of each index was calculated using R4.03 software, and the Kaplan-Meier(K-M) survival curve was drawn. Nomogram software was used for validation. *P* values < 0.05 were considered statistically significant.

## Results

### Clinicopathological features of LUAD patients

Our analysis included 89 patients with L858R mutations and 196 patients with 19DEL mutations, respectively, with a mean age of 58.67 ± 9.30 years and 58.01 ± 9.01 years, respectively. Among the 89 patients with L858R mutation, 11 (12.4%) were stage III, 80 (85.4%) were stage IV, 69 (77.5%) received chemotherapy, and 84 (94.4%) received targeted therapy. Among the 196 patients with 19DEL mutations, 25 (12.8%) were stage III, 161 (82.1%) were stage IV, 159 (81.1%) received chemotherapy, and 181 (92.3%) received targeted therapy. The clinicopathological data are shown in Table 2.

**Table 2:** Baseline on L858R and 19DEL categorical variables in our study

Features	L858R	19DEL
**Total**	89	196
**Gender**		
Male (%)	44 (49.4%)	105(53.6%)
Female (%)	45 (50.6%)	91(46.4%)
**Age (years**, X ± SD)	58.67 ± 9.30	58.01 ± 9.01
**TNM stage**		
Stage I (%)	1 (1.1%)	6 (3.1%)
Stage II (%)	1 (1.1%)	4 (2.0%)
Stage III (%)	11 (12.4%)	25 (12.8%)
Stage IV (%)	80 (85.4%)	161 (82.1%)
**T stage**		
T1 (%)	16 (18.0%)	38 (19.4%)
T2 (%)	19 (21.3%)	52 (26.5%)
T3 (%)	13 (14.6%)	27(13.8%)
T4 (%)	41 (46.1%)	79(40.3%)
**N stage**		
0 (%)	7 (7.9%)	23 (11.7%)
1 (%)	8 (9.0%)	32 (16.3%)
2 (%)	51 (57.3%)	88 (45.0%)
3 (%)	23 (25.8%)	53 (27.0%)
**M**		
0 (%)	13 (14.6%)	34 (17.3%)
1 (%)	76 (85.4%)	162 (82.7%)
**Chemotherapy**		
Yes (%)	69 (77.5%)	159 (81.1%)
No (%)	20 (22.5%)	37 (18.9%)
**Targeted therapy**		
Yes (%)	84 (94.4%)	181 (92.3%)
No (%)	5 (5.6%)	15 (7.7%)
eHSP90α (median [IQR])	40.46 [28.82,65.15]	45.60[31.01,72.85]
T lymphocytes (median [IQR])	64.30 [55.75,72.22]	62.69[56.54,71.23]
T helper cells (median [IQR])	33.10 [29.69,40.42]	35.60[30.75,41.70]
suppressor T cells (median [IQR])	23.00 [16.65,27.65]	21.29[16.80,25.30]
T helper cells/ suppressor T cells (median [IQR])	1.50 [1.20,2.10]	1.70[1.30,2.30]
Natural killer cells (median [IQR])	15.10 [9.55,21.95]	14.60[9.23,20.88]
B lymphocytes (median [IQR])	9.70 [6.00,15.35]	11.10[7.45,15.40]
Carcinoembryonic antigen (median [IQR])	14.22 [2.83,52.92]	17.73[3.37,120.75]
Carcinoembryonic antigen 125(median [IQR])	34.60[17.20,92.90]	41.80[17.28,187.10]
Carcinoembryonic antigen 153(median [IQR])	19.50[10.40,33.00]	23.40[13.38,50.03]
Carbohydrate antigen 199 (median [IQR])	7.60 [8.00,26.70]	7.75[2.18,36.20]
Thymidine kinase1 (median [IQR])	0.77[0.30,1.22]	0.69[0.36,1.35]

Abbreviation: CEA: carcinoembryonic antigen; CA125: carbohydrate antigen 125; CA153: carbohydrate antigen 153; CA199: carbohydrate antigen 199; eHSP90α: extracellular heat shock protein 90 AA1; T cell: T lymphocytes; Th: T helper cells; Ts: suppressor T cells; B cell: B lymphocytes; Th/Ts: T helper cells/ suppressor T cells; NK: natural killer; TK1: thymidine kinase 1

Furthermore, we analyzed the K-M survival curves of OS for two mutations and tested them with log rank. The K-M survival curve shows that patients with 19DEL mutation predicted a worse prognosis (Fig. 1D), and the median survival time of L858R mutation patients was 43 months; the median survival time of patients with 19DEL mutation was 63 months. Also, statistics show that the OS of L858R patients was lower than that of 19DEL patients (Fig. 1C).

### Correlations of eHSP90α and clinical index

CEA is the most common tumor marker for LUAD, and we analyzed the correlation between eHSP90α and CEA in LUAD and with other clinical biomarkers (Table 3). Through analysis, we found that eHSP90α was positively correlated with CEA, CA125, and CA153 among all LUAD patients we recruited. When differentiating mutations, eHSP90α was only positively associated with CEA in patients with L858R mutation. In patients with 19DEL mutation, eHSP90α was positively correlated with CEA, CA125, and CA153. eHSP90α may have good diagnostic and prognostic value, but there may also be some differences between the two mutations.

**Table 3:** Correlation of eHSP90α and other Index in LUAD patients

Parameter	Total		L858R		19DEL	
	*r*	*p*	*r*	*p*	*r*	*p*
Sex	-0.138	**0.029**	-0.288	**0.011**	-0.076	0.319
Age	-0.132	**0.038**	-0.002	0.986	-0.178	**0.020**
T lymphocytes	0.059	0.375	-0.003	0.978	0.086	0.288
T helper cells	0.078	0.247	0.070	0.565	0.081	0.318
suppressor T cells	-0.032	0.631	-0.059	0.625	-0.004	0.962
T helper cells/ suppressor T cells	0.100	0.135	0.112	0.355	0.078	0.338
Natural killer cells	-0.083	0.212	-0.079	0.516	-0.062	0.441
B lymphocytes	0.002	0.981	-0.097	0.426	0.028	0.733
CEA	0.324	**0.001**	0.270	**0.021**	0.340	**0.001**
CA125	0.289	**0.001**	0.226	0.055	0.315	**0.001**
CA153	0.206	**0.002**	0.199	0.091	0.210	**0.008**
CA199	0.119	0.069	0.158	0.183	0.083	0.299
TK1	-0.026	0.690	-0.004	0.976	-0.007	0.934

Abbreviation: CEA: carcinoembryonic antigen; CA125: carbohydrate antigen 125; CA153: carbohydrate antigen 153; CA199: carbohydrate antigen 199; eHSP90α: extracellular heat shock protein 90 AA1; T cell: T lymphocytes; Th: T helper cells; Ts: suppressor T cells; B cell: B lymphocytes; Th/Ts: T helper cells/ suppressor T cells; NK: natural killer; TK1: thymidine kinase 1

### Diagnostic value of biomarkers in LUAD

To better study the influence of clinical and biological indicators on the prognosis of LUAD patients, we made Receiver operating characteristic(ROC) curves of each physical index with OS as the outcome. We calculated the area under the curve(AUC) to judge its diagnostic efficiency (Table 4). Through analysis, we found that the top three AUC values in all our included patient data were CEA, CA125, and eHSP90α, with 0.718, 0.668, and 0.644, respectively. In L858R mutant LUAD patients, the top three places calculated by the area under the curve were eHSP90α, CEA, and CA125, and their AUC values were 0.765, 0.682, and 0.63, respectively. In patients with 19DEL mutation, the top 3 areas under the curve were CEA, CA125, and CA153, and their AUC values were 0.734, 0.684, and 0.673, respectively, while eHSP90α had an AUC of 0.591 and a Youden index of 0.209. Suppose a single biomarker is used to diagnose LUAD among the L858R mutations. In that case, the highest specificity is CEA, the highest sensitivity is Ts, and the highest Youden index is eHSP90α, with a Youden index of 0.505. In contrast, among the 19DEL mutations, the highest specificity is T cell, the highest sensitivity is CA.153, and the highest Youden index is CEA, with a Youden index of 0.397. So, the CEA index has the best diagnostic value in patients with 19DEL mutation, while eHSP90α has a better diagnostic value in L858 mutation patients in LUAD.

**Table 4:** Receiver operating characteristic curve of parameters

Characteristics	Total					L858R					19DEL				
	AUC (95%CI)	Threshold	specificity	Sensitivity	Youden	AUC (95%CI)	Threshold	specificity	Sensitivity	Youden	AUC (95%CI)	threshold	specificity	sensitivity	Youden
CEA	**0.716(0.65–0.783)**	45.82	0.804	0.573	**0.378**	0.682(0.557–0.807)	41	**0.804**	0.556	0.359	**0.734(0.657–0.811)**	46.815	0.797	0.6	**0.397**
CA125	0.668(0.598–0.738)	43.9	0.626	0.646	0.272	0.63(0.502–0.758)	55.8	0.714	0.556	0.27	0.684(0.599–0.769)	38.6	0.585	0.745	0.331
eHSP90α	0.644(0.57–0.718)	35.6	0.45	0.825	0.275	**0.765(0.649–0.88)**	44.8	0.755	0.75	**0.505**	0.591(0.499–0.683)	35.75	0.388	0.821	0.209
CA153	0.639(0.569–0.71)	18.85	0.508	0.744	0.252	0.577(0.445–0.709)	19	0.554	0.667	0.22	0.673(0.591–0.755)	12.5	0.317	**0.982**	0.299
Natural killer cells	0.592(0.512–0.672)	19.25	0.756	0.429	0.184	0.538(0.4-0.676)	16.865	0.625	0.56	0.185	0.612(0.514–0.71)	24.635	0.9	0.346	0.246
CA199	0.588(0.508–0.668)	30.1	0.827	0.415	0.241	0.573(0.44–0.707)	14.2	0.643	0.519	0.161	0.592(0.49–0.693)	30.1	0.829	0.455	0.284
B lymphocytes	0.568(0.493–0.643)	11.8	0.477	0.688	0.166	0.467(0.334–0.601)	6.1	0.286	0.8	0.086	0.586(0.494–0.677)	12.085	0.492	0.712	0.203
TK1	0.54(0.466–0.614)	0.305	0.258	**0.877**	0.135	0.566(0.427–0.704)	0.63	0.473	0.704	0.176	0.531(0.444–0.618)	0.305	0.252	0.926	0.178
T helper cells	0.538(0.459–0.616)	34.25	0.562	0.545	0.108	0.58(0.447–0.713)	36.415	0.714	0.52	0.234	0.587(0.489–0.685)	34.25	0.642	0.577	0.219
T lymphocytes	0.533(0.454–0.611)	51.965	**0.875**	0.234	0.109	0.504(0.374–0.634)	60.25	0.393	0.8	0.193	0.552(0.454–0.651)	52.015	**0.883**	0.308	0.191
suppressor T cells	0.508(0.433–0.584)	26.165	0.278	0.844	0.123	0.525(0.394–0.656)	26.15	0.375	**0.84**	0.215	0.5(0.406–0.594)	18.5	0.383	0.731	0.114
T helper cells/ suppressor T cells	0.48(0.402–0.558)	1.65	0.511	0.532	0.044	0.547(0.411–0.683)	1.35	0.411	0.76	0.171	0.548(0.451–0.645)	1.55	0.642	0.462	0.103

Abbreviation: CEA: carcinoembryonic antigen; CA125: carbohydrate antigen 125; CA153: carbohydrate antigen 153; CA199: carbohydrate antigen 199; eHSP90α: extracellular heat shock protein 90 AA1; T cell: T lymphocytes; Th: T helper cells; Ts: suppressor T cells; B cell: B lymphocytes; Th/Ts: T helper cells/ suppressor T cells; NK: natural killer; TK1: thymidine kinase 1; OS: overall survival; PFS: progression-free survival

Note: Bold font indicates maximum value

### Value of eHSP90α in clinical prognostic assessment in patients with L858R mutation and 19DEL mutation

Then, we explored the different expressions of eHSP90α in Alive and Dead groups in OS in patients with L858R and 19DEL mutations, respectively. Statistics show that in L858 patients, those who eventually died had higher eHSP90α levels than the non-dead group, whereas in 19del, there was no difference in eHSP90α levels between the two groups (Fig. 1B).

**Fig. 2:**
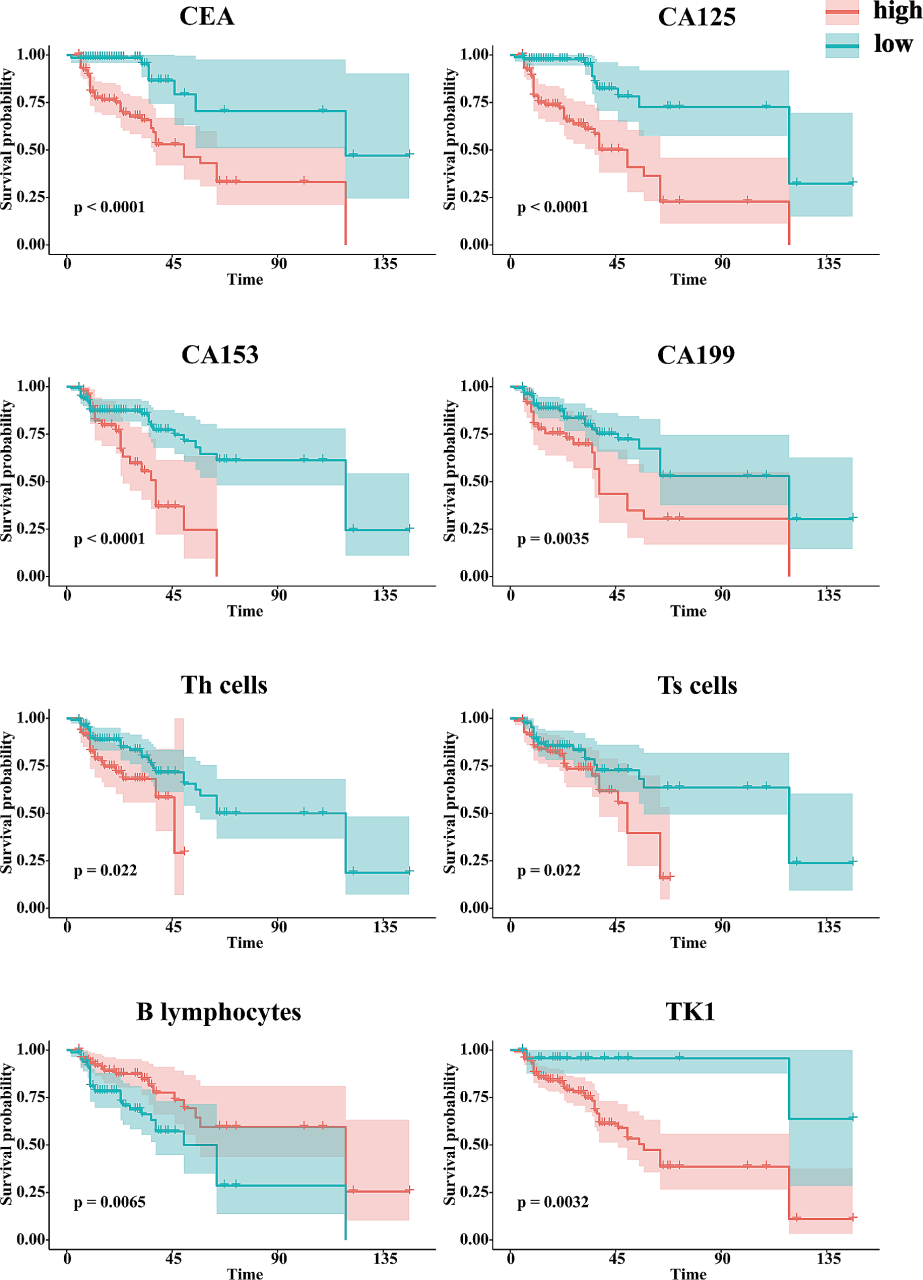
Kaplan-Meier Survival curve of CEA, CA125, CA153, CA199, Th cells, Ts cells, B lymphocytes, TK1 of OS in patients with LUAD 19DEL mutation

The K-M survival analysis was performed to determine the cutoff points of the index. The cut-off value of eHSP90α for L858R mutation with OS was 44.5ng/mL, and for 19DEL mutation was 40.8ng/mL. Kaplan-Meier survival analysis showed that L858R mutation patients with high eHSP90α levels had poor OS. However, this trend could not be detected in19DEL mutation patients (Fig. 1E). Other clinical biomarkers were also subjected to K-M survival analysis, and the indicators that have predictive value on prognosis are shown in Figs. 1E and 2.

**Fig. 3:**
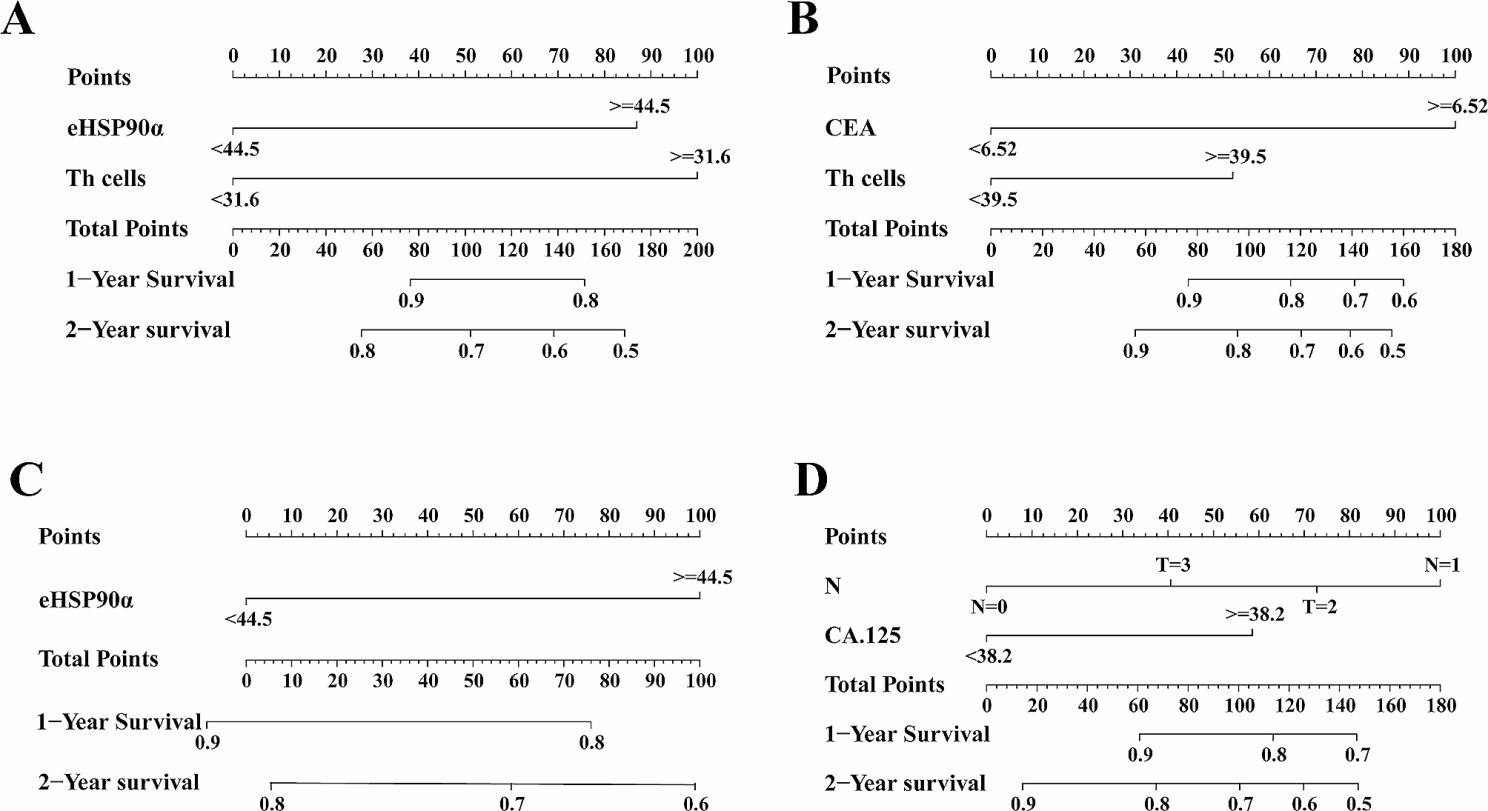
Nomograms based on OS in patients with LUAD 19DEL and L858R mutations. The prognostic nomogram for OS is based on the prognostic scores of eHSP90α and other factors in (**A**) L858R and (**C**)19DEL patients. The prognostic nomogram for PFS is based on the prognostic scores of eHSP90a and other factors in (**B**) L858R and (**D**) 19DEL patients

We performed COX univariate and multivariate analyses for OS outcomes to determine which factors are independent in the prognosis of patients with L858R and 19DEL mutation (Tables 5 and 6). In multivariate analysis, we found that in patients with L858R mutation, high eHSP90α expression and high Th levels were short OS significantly associated (eHSP90a, *P* = 0.0305 and Th, *P* = 0.0282); In patients with 19DEL mutations, high CEA and Th levels had independent indicators of the prognosis of their short OS (CEA, *P* = 0.0150, and Th, *P* = 0.0392). Therefore, we included eHSP90α and Th, CEA, and Th as reference factors for two different mutations, constructing their nomograms to predict the OS of two other mutations (Fig. 3).

**Table 5:** Cox regression analysis on the L858R OS and PFS of LUAD

Variables	Univariate Cox regression analysis						Multivariate Cox regression analysis					
	OS			PFS			OS			PFS		
	*P*-value	HR	95%CI	*P*-value	HR	95%CI	*P*-value	HR	95%CI	*P*-value	HR	95%CI
Stage												
Stage II	1.000	9.840e-01	0-Inf	1.000	9.598e-01	0-Inf						
Stage III	0.998	1.886e + 07	0-Inf	0.998	1.021e + 07	0-Inf						
Stage IV	0.998	2.623e + 07	0-Inf	0.997	3.063e + 07	0-Inf						
T stage												
T2	0.578	0.6279	0.1221-3.230	0.729	1.2252	0.3878–3.871						
T3	0.419	1.8324	0.4213–7.969	0.381	1.7083	0.5156-5.660						
T4	0.271	2.0150	0.5794–7.008	0.347	1.6295	0.5893–4.505						
N stage												
N1	0.349	0.3171	0.0287–3.507	0.996	7.203e + 07	0-Inf						
N2	0.289	2.2549	0.5021–10.124	0.996	9.526e + 07	0-Inf						
N3	0.256	2.4808	0.5167–11.911	0.996	8.516e + 07	0-Inf						
M stage	0.486	1.6799	0.3899–7.237	0.0667	3.8515	0.9111–16.28						
eHSP90α	**0.0371**	2.7732	1.063–7.237	**0.0156**	2.4257	1.183–4.976	**0.0305**	2.3846	1.085–5.239	**0.0173**	2.4126	1.168–4.982
TK1	0.194	1.9369	0.7137–5.256	0.602	1.3773	0.4129–4.594						
CEA	0.516	0.7094	0.2517–1.999	0.213	1.9738	0.677–5.754						
CA125	0.604	0.7845	0.3132–1.965	0.168	1.8826	0.7657–4.629						
CA153	0.0805	2.0037	0.9192–4.368	**0.0474**	2.0159	1.008–4.031						
CA199	0.165	1.8223	0.7817–4.248	0.124	1.9397	0.833–4.517						
T cell	0.0931	2.3224	0.8686-6.21	0.266	1.5730	0.708–3.494						
Th	**0.0195**	2.9964	1.194–7.522	0.411	1.3988	0.6284–3.114	**0.0282**	2.7168	1.112–6.635			
Ts	0.218	3.537	0.473–26.45	0.125	3.0922	0.7303–13.09						
Th/Ts	0.174	1.8946	0.7538–4.762	0.247	0.6552	0.3204-1.34						
NK cell	0.13	0.4214	0.1376–1.291	0.572	0.7347	0.2522-2.14						
B cell	0.263	0.4370	0.1026–1.861	0.635	0.7742	0.2691–2.228						

Abbreviation: CEA: carcinoembryonic antigen; CA125: carbohydrate antigen 125; CA153: carbohydrate antigen 153; CA199: carbohydrate antigen 199; eHSP90α: extracellular heat shock protein 90 AA1; T cell: T lymphocytes; Th: T helper cells; Ts: suppressor T cells; B cell: B lymphocytes; Th/Ts: T helper cells/ suppressor T cells; NK: natural killer; TK1: thymidine kinase 1; OS: overall survival; PFS: progression-free survival; CI: confidence interval; HR: hazard ratio; OS: overall survival; PFS: progression-free survival

**Table 6:** Cox regression analysis on the 19DEL OS and PFS of LUAD

Variables	Univariate Cox regression analysis						Multivariate Cox regression analysis					
	OS						PFS					
	*P*-value	HR	95%CI	*P*-value	HR	95%CI	*P*-value	HR	95%CI	*P*-value	HR	95%CI
Stage												
Stage II	0.997	3.662e + 07	0-Inf	0.994	4.437e + 07	0-Inf						
Stage III	0.997	3.662e + 07	0-Inf	0.994	2.128e + 07	0-Inf						
Stage IV	0.997	2.749e + 07	0-Inf	0.994	2.745e + 07	0-Inf						
T stage												
T2	0.898	0.95327	0.4591–1.979	**0.0297**	1.97552	1.0695–3.649						
T3	0.578	0.76585	0.4591–1.979	0.3677	1.39973	0.6734–2.909						
T4	0.835	0.92570	0.4468–1.918	0.8455	0.94055	0.5078–1.742						
N stage												
N1	0.248	3.364	0.4289–26.38	**0.00188**	5.5057	1.8785–16.137				**0.03526**	3.2972	1.0859–10.012
N2	0.219	3.529	0.4719–26.39	**0.01716**	3.4622	1.2468–9.614						
N3	0.111	3.529	0.6855–39.33	0.05054	2.8848	0.9975–8.343						
M stage	0.244	1.6591	0.7079–3.889	0.129	1.5559	0.879–2.754						
eHSP90α	0.0581	1.7540	0.981–3.136	0.192	1.3711	0.8532–2.203						
TK1	**0.029**	4.8301	1.174–19.86	0.407	0.7786	0.4308–1.407						
CEA	**3.18e-05**	5.1159	2.371–11.04	**0.00457**	1.9651	1.232–3.134	**0.0150**	3.4321	1.2701–9.274			
CA125	**2.78e-06**	4.8388	2.502–9.357	**0.000337**	2.2489	1.444–3.503				**0.00575**	2.1283	1.2452–3.638
CA153	**0.000146**	3.0534	1.716–5.432	0.257	1.3070	0.8227–2.076						
CA199	**0.00833**	2.0538	1.203–3.506	0.416	1.2088	0.7655–1.909						
T cell	0.078	1.7699	0.938–3.34	0.286	1.3013	0.8022–2.111						
Th	**0.0345**	1.9786	1.051–3.725	0.0803	0.6525	0.4044–1.053	**0.0392**	2.0958	1.0373–4.234			
Ts	**0.0413**	1.8481	1.025–3.334	0.0704	1.5022	0.9667–2.334						
Th/Ts	0.219	0.6620	0.343–1.278	**0.0209**	0.5316	0.311–0.9088						
NK cell	0.453	0.7758	0.3998–1.505	0.252	1.3675	0.8004–2.337						
B cell	**0.0131**	0.4836	0.2724–0.8586	0.11	0.6990	0.4508–1.084						

Abbreviation: CEA: carcinoembryonic antigen; CA125: carbohydrate antigen 125; CA153: carbohydrate antigen 153; CA199: carbohydrate antigen 199; eHSP90α: extracellular heat shock protein 90 AA1; T cell: T lymphocytes; Th: T helper cells; Ts: suppressor T cells; B cell: B lymphocytes; Th/Ts: T helper cells/ suppressor T cells; NK: natural killer; TK1: thymidine kinase 1; OS: overall survival; PFS: progression-free survival; CI: confidence interval; HR: hazard ratio; OS: overall survival; PFS: progression-free survival

### Indicator value of eHSP90α in PFS and response evaluation in patients with L858R mutation and 19DEL mutation

Compared with OS, PFS and treatment response (RECIST version 1.1) reflect the short-term survival benefits of patients more. Therefore, PFS was first used as the outcome for univariate and multivariate analysis of Cox (Tables 5 and 6). In multivariate analysis, we found a significant relationship between high eHSP90α expression and short PFS in patients with L858R mutation (eHSP90α, *P* = 0.0173). In patients with 19DEL mutations, high CA125 is an independent indicator of the prognosis of PFS (CA125, *P* = 0.000575).

**Fig. 4:**
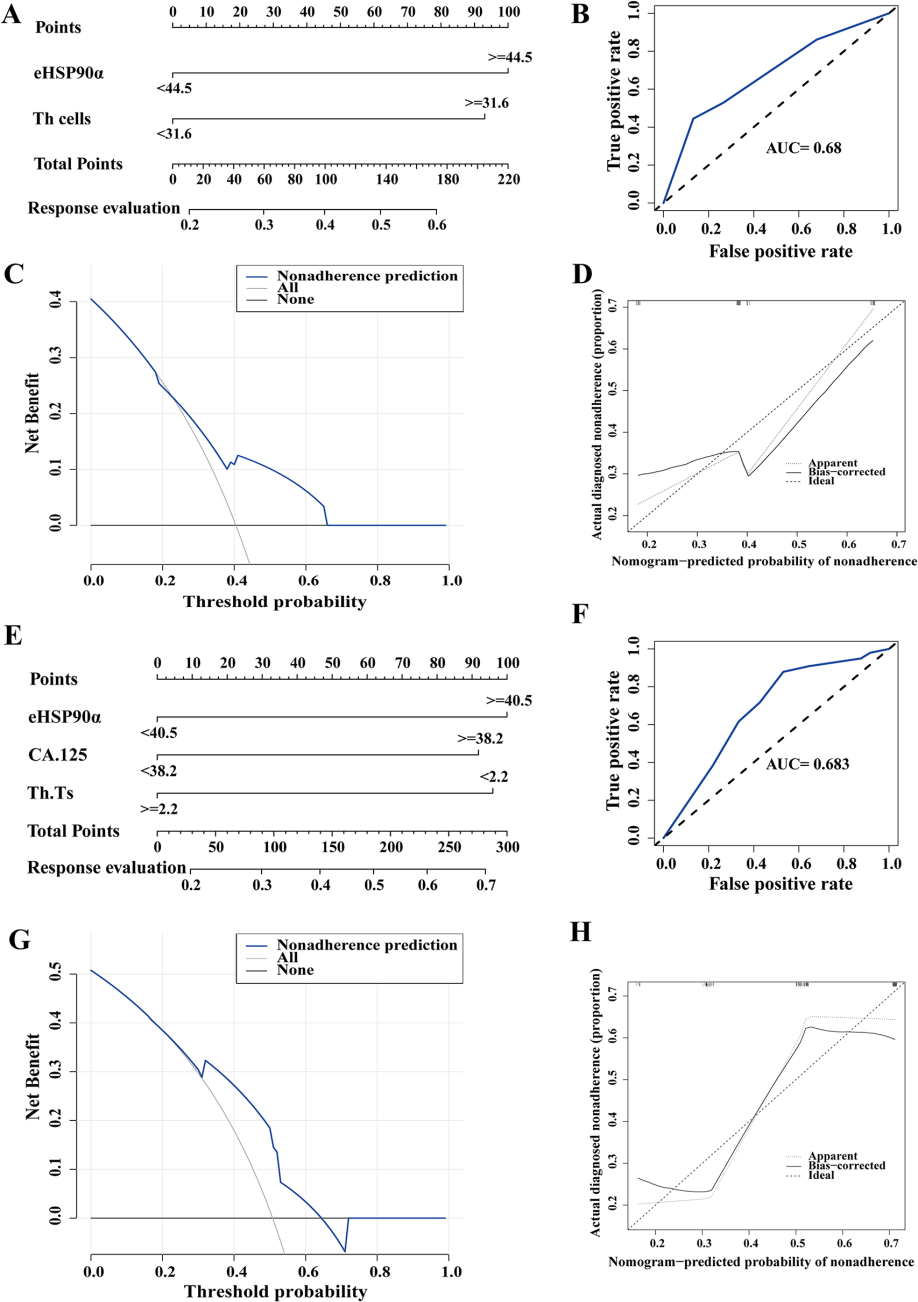
The predictive value of eHSP90α in treating patients with L858R and 19DEL mutations in LUAD. Established (**A**) L858R and (**E**) 19DEL mutation response evaluation nomogram with eHSP90α. Receiver operating characteristic curve of (**B**) L858R and (**F**) 19DEL model. The decision curve analysis of (**C**) L858R and (**G**) 19DEL models showed a response evaluation nomogram. The X-axis is the risk threshold probability that changes from 0 to 1, and the Y-axis is the calculated net profit for a given threshold probability. Calibration curves of (**D**) L858R and (**H**) 19DEL nomogram. Notes: The x-axis represents the predicted probability of progression. The y-axis represents the actual progression of small-cell lung cancer. The diagonal dotted line represents a perfect prediction by an ideal model. The solid line represents the performance of the nomogram, of which a closer fit to the diagonal dotted line means a better prognosis

Then, eHSP90a was used to explore the diagnostic significance of response status. We screened 89 L858R mutant patients and 196 patients with 19DEL, using LASSO and 10-fold cross-validation, respectively, which were stage, M stage, eHSP90α, Th, Ts (lambda, Min = 0.0472479) and stage, eHSP90α, CEA, CA125, Th, Th.Ts (lambda, Min = 0.04348617). Multiple logistic regression analysis was performed on the above features to further select the independent variables of effective chemotherapy from the LASSO results. The L858R mutation data model ultimately containede HSP90α(OR = 2.865121e + 00, *p* = 0.0324) and Th(OR = 3.182332e + 00, *p* = 0.0288), and the 19DEL mutation data model ultimately contained eHSP90α (OR = 2.130448e + 00, *p* = 0.0246), CA.125 (OR = 2.087725e + 00, *p* = 0.0367), Th.Ts (OR = 4.334531e-01, *p* = 0.0260) (Fig. 4A adn 4E). The AUC of the model in L858R mutation and 19DEL were 0.68 and 0.683, respectively (Fig. 4B and F). The models were verified by bootstrapping (Fig. 4D, C-index = 0.68 and 4 H, C-index = 0.683). In this study, the decision curve analysis of two models showed that the response evaluation nomogram would profit more than the threshold (Fig. 4C and G).

## Discussion

EGFR is a transmembrane glycoprotein distributed on the surface of epithelial cells and consists of extracellular ligand-binding regions, transmembrane regions, and intracellular tyrosine kinase binding regions. Studies have found that patients with EGFR gene mutations are relatively more likely to have metastasis. The most common is bone metastasis and brain metastasis [16]. In recent years, targeted therapy for patients with EGFR mutations has achieved significant clinical efficacy, significantly prolonging survival compared with traditional chemotherapy regimens [17]. 19DEL and L858R are the two most common sensitive mutations in EGFR-TKI. Data from multiple trials suggest that the efficiency of different treatment strategies may vary depending on various EGFR mutation states, particularly between 19DEL and L858R mutations. For example, studies have found that patients with 19DEL have a higher PFS than patients treated with TKIs than patients with L858R [18]. It has also been found that OS in patients with 19DEL is higher than in patients with L858R in our study [7].

HSP90α is the only isoform detected in plasma. In addition to participating in various cell life activities such as apoptosis, it has also been used as a clinical biomarker in multiple cancers. Several studies have shown that it has good diagnostic performance and prognostic value as a marker for cancers [19–21]. Studies have long found that eHSP90a can assist in diagnosing LC and is associated with its progression [14, 22]. Still, no one has explored the expression and diagnostic efficacy of eHSP90a in different subtypes of EGFR mutations in LUAD. Our previous study found that in SCLC, the diagnostic efficacy of eHSP90 as a prognostic evaluation and diagnostic marker is better than NSE, a biomarker of classical SCLC [15]; In this study, we found a positive correlation between eHSP90α and CEA. In the evaluation of diagnostic efficacy, we found that among the patients with L858R mutation, eHSP90α had the best diagnostic efficacy (AUC = 0.765) among all the indicators we included in the evaluation and was only slightly inferior to CEA in a single specific index; In the data of patients with 19DEL mutation, we found that the performance of eHSP90α is somewhat insufficient, and the biological indicator with the highest diagnostic efficacy is CEA (AUC = 0.734), despite this, CEA is still not as sensitive as eHSP90α. Another study also found that eHSP90α is a valuable predictor of response to early chemotherapy and positively correlated with tumor remission after chemotherapy in NSCLC [23]. This study found that high eHSP90α was closely related to short OS, PFS, and progression after treatment in L858R mutation patients. Among 19DEL mutation patients, high eHSP90α can only predicted poor treatment response. eHSP90α is a highly sensitive pan-cancer marker; its specificity may not be enough, so this paper for two different mutation types, using eHSP90α as the basis, combined with other markers to construct their corresponding prognostic models.

This study also found that high Th cells were closely related to poor OS in 19DEL and L858R mutation patients. Previous works of literature found that NSCLC patients in the advanced group were evidently lower in the expression of CD4 + but markedly higher in the expression of CD8 + in peripheral blood than in the early group [24]. Another study found that LUAD has higher CD4 + expression than other subtypes in NSCLC [24, 25]. Moreover, high expression of CD4 + may indicate a better immune response and should indicate better patient survival. Thus, the mechanism still needs to be explored.

In summary, this study found that patients with L858R mutation had worse OS than those with 19DEL mutation. In patients with L858R mutation, eHSP90α is closely related to short OS, PFS, and progression after treatment. As a new biomarker, eHSP90α is highly valued in evaluating its prognosis. However, in patients with 19DEL mutation, the indicative value of eHSP90α is relatively limited, and it only has a specific, meaningful value for the response to treatment. However, this study also had some limitations. First, this is a single-center study; the data may had some selection bias. Second, the accuracy of our nomogram should be evaluated through external validation, which will help to assess whether our nomogram applies to new Populations. Large-scale clinical trials are needed to illustrate and improve the model’s effectiveness in determining prognosis in L858R and 19DEL mutant patients. Finally, we will refine the diagnostic models and cost-effectiveness analyses and improve their clinical utility.

### Electronic supplementary material

Below is the link to the electronic supplementary material.

Supplementary Material 1

## Data Availability

This published article and its supplementary information files include all data generated or analyzed during this study. The corresponding author can provide all possible assistance to the requester of the original data.
