# Supplementary material for: eHSP90α in front-line therapy in EGFR exon 19 deletion and 21 Leu858Arg mutations in advanced lung adenocarcinoma
Source: BMC Cancer. 2024 Jul 12;24:835. doi: 10.1186/s12885-024-12573-3 (PMC11245848; doi:10.1186/s12885-024-12573-3)
Supplement: Supplementary file 1 — Supplementary Material 1 [file 12885_2024_12573_MOESM1_ESM.docx]

Supplementary Material

**Supplementary Figure 1.**


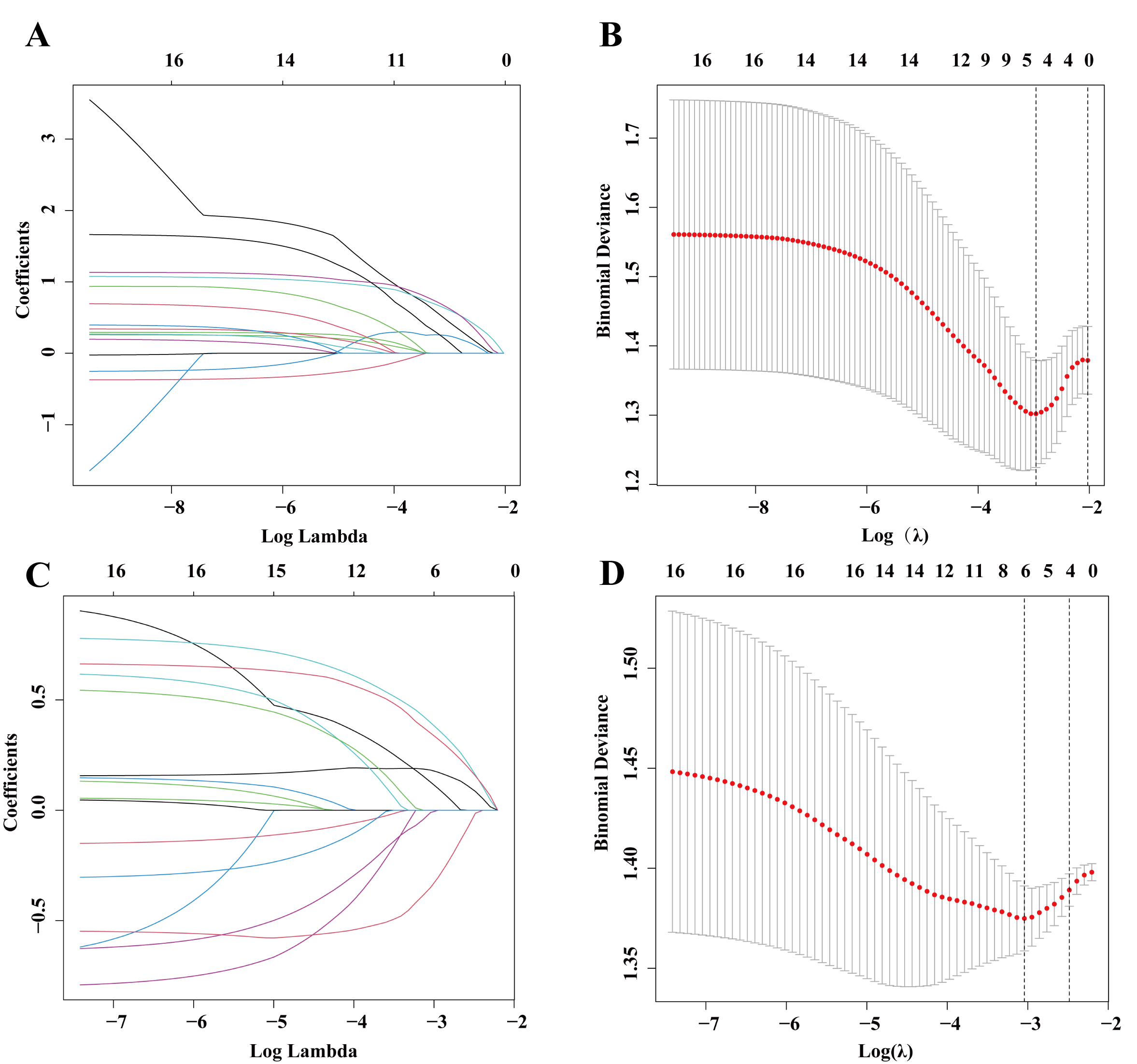


**Predictive value of eHSP90a in the treatment of patients with** **L858R and 19DEL mutations in LUAD** LASSO coefficient profiles of the 16 features in (A) L858R and (C) 19DEL mutations. A coefficient profile plot was produced against the logλ sequence in (B) L858R and (D) 19DEL mutations
